# The Relationship Between Sexual Dysfunctions With Disease Prolactin and Genetic Polymorphisms in Schizophrenia and Bipolar Disorder Patients Receiving Pharmacotherapy

**DOI:** 10.7759/cureus.60654

**Published:** 2024-05-20

**Authors:** Neşe Öztürk Atkaya, İsmail Osman Özdel, Samet Türel, Emre Tepeli

**Affiliations:** 1 Department of Psychiatry, Faculty of Medicine, Pamukkale University, Denizli, TUR; 2 Department of Medical Genetics, Faculty of Medicine, Pamukkale University, Denizli, TUR

**Keywords:** genetics, psychiatry, sexual dysfunction, bipolar disorder, schizophrenia

## Abstract

Objective: The aim of this study is to investigate sexual dysfunctions (SDs) and related factors in patients with schizophrenia and bipolar disorder receiving pharmacotherapy.

Methods: This study included 111 patients. The Scale for the Assessment of Positive Symptoms (SAPS), the Scale for the Assessment of Negative Symptoms (SANS), and the Calgary Depression Scale for Schizophrenia (CDSS) were applied to the schizophrenia, and the Young Mania Rating Scale (YMRS) and Hamilton Depression Rating Scale (HAM-D) to the bipolar patient group. The sociodemographic data form and the Arizona Sexual Experiences Scale (ASEX) were applied to both of the patient groups. Blood was drawn from all patients to evaluate the indicated gene polymorphisms and evaluate prolactin levels.

Results: SD was detected in 45.9% (N = 34) of the schizophrenia group, and 59.5% (N = 22) in the bipolar disorder group. SD was significantly higher in elderly patients and patients with a high smoking amount and low education levels. The eNOS -786T>C T allele frequency was found to be significantly higher in patients with SD. The logistic regression analysis determined that eNOS -786T>C CT and TT genotypes increased the risk of SD.

Conclusion: In this study, the high rates of SD in patients with schizophrenia and bipolar disorder, and the presence of modifiable factors that influence the presence of SD, suggest that SD should be given more attention in these patient groups. On the other hand, the high rate of SD in patients with the eNOS -786T>C T allele indicates the importance of carrying out new studies investigating the factors affecting the enzyme activity in this genotype. There is a need for more studies on eNOS genotypes and enzyme activites in this area.

## Introduction

Schizophrenia and bipolar disorder are psychiatric disorders that cause significant disability, the course and outcome of which vary from patient to patient and over time. Sexual dysfunction (SD) in schizophrenia and bipolar disorder may develop because of factors such as clinical symptoms of the disease, neuroendocrine changes in the disease, drug side effects, or lack of social skills [1-3].

SD may lead to deterioration in a patient’s quality of life, deterioration of drug compliance, dropouts from treatment, and worsening of the underlying psychiatric illness.

The aim of this study, which was based on SDs in schizophrenia and bipolar disorder patient groups under pharmacotherapy, was to investigate the relationship between SD and genetic polymorphisms (eNos, nNos, DRD2-141CIns/Del, DRD2Taq1A polymorphism), prolactin levels, and diseases to shed light on the use of drugs, diseases, genetics, and individual factors as predictors of sexual side effects in patients receiving pharmacotherapy.

## Materials and methods

The research was conducted between January 2014 and August 2015 in Pamukkale University Faculty of Medicine, Department of Psychiatry.

The patients who applied to Pamukkale University Faculty of Medicine, Department of Psychiatry, and were diagnosed with bipolar disorder and schizophrenia according to the Diagnostic and Statistical Manual of Mental Disorders, Fourth Edition, Text Revision (DSM-IV-TR) (and also the fifth edition), were included in this study. The sociodemographic data form and the Arizona Sexual Experiences Scale (ASEX) were applied to both of the patient groups. The Scale for the Assessment of Positive Symptoms (SAPS), the Scale for the Assessment of Negative Symptoms (SANS), the Calgary Depression Scale for Schizophrenia (CDSS), the Young Mania Rating Scale (YMRS), and the Hamilton Rating Scale for Depression (HAM-D) were applied according to the patient groups. Blood was drawn to measure patients’ blood prolactin levels and to examine the indicated genetic polymorphisms.

The inclusion criteria for the patient group were patients diagnosed with schizophrenia or bipolar disorder according to DSM IV-TR diagnostic criteria (and also the fifth edition) who are literate, getting pharmacotherapy at least for six months, did not change the pharmacotherapy at least for the last two months, were at remission or partial remission and were between 18-65 years of age. The patients gave consent for the study after they were told about the aim and procedure of the study.

The exclusion criteria for the patient group were the presence of hypertension, diabetes mellitus, neurological disorders, urological disorders, mental retardation, active findings, or impairment, which preclude performing a clinical evaluation, presence of a psychiatric disorder associated with a general medical condition, and the presence of alcohol or substance use disorder except smoking.

All volunteers participating in the study were informed about the study in accordance with the Declaration of Helsinki, and their written consents were obtained. Prior to the research, the approval of the Pamukkale University Clinical Research Ethics Committee dated 30/12/2013 and numbered 2013TPF028 was obtained. The local Ethics Committee has approved the project, and the work that was undertaken conforms to the provisions of the Declaration of Helsinki. Written informed consents of the patients were obtained.

Sociodemographic data form

It is an information form prepared by us to collect sociodemographic data of the cases.

The ASEX

It is a self-report scale developed by McGahuey et al. [4]. It is a six-point Likert-type scale that consists of five items questioning the areas such as sexual desire, arousal, erection/vaginal wetting, reaching orgasm, and getting satisfaction from orgasm. It has separate forms for men and women. The reliability study of the Turkish form was performed by Soykan [5].

The SAPS

It provides a six-point Likert-type assessment including hallucinations, delusions, strange behavior, and structural thought disorder [6]. It consists of four subscales and 34 items. The total score varies between 0-170. The validity and reliability study of the Turkish version of the scale was performed by Erkoc et al. [7].

The SANS

It aims to measure the level, distribution, and severity of negative symptoms of schizophrenia. It was developed by Andreasen et al. [6]. It includes five subscales and twenty-five items that are evaluated by the interviewer, providing a six-point Likert-type measurement. The validity and reliability study for the Turkish version of the scale was performed by Erkoc et al. [8].

The CDSS

It is a scale developed by Addington et al. [9]. It consists of nine items that provide a four-point Likert-type measurement evaluated by the interviewer. Each item receives scores ranging from 0-3, and the total score is obtained by summing them up. The total score ranges from 0 to 27. The validity and reliability study for the Turkish version of the scale was done by Oksay et al. [10].

The HAM-D

It is applied to determine the level of depression, the change in severity, and the symptom pattern in the patient. It was developed by Hamilton [11]. It is applied by the clinician and consists of 17 items. The validity and reliability study for the Turkish form of the scale was conducted by Akdemir et al. [12].

The YMRS

It was developed by Young et al. to measure the severity and change of the manic state [13]. It consists of 11 items, seven of which are five-point Likert type and four are nine-point Likert type questions. The validity and reliability study for the Turkish version of the scale was conducted by Karadag et al. [14].

Statistical analysis

The data were analyzed using the Statistical Product and Service Solutions (SPSS) (IBM SPSS Statistics for Windows, Armonk, NY) package program. Continuous variables were given as mean ± standard deviation and categorical variables as numbers and percentages. When parametric test assumptions were met, the significance test of the difference between two means and ANOVA was used to compare independent group differences, and when parametric test assumptions were not met, the Mann-Whitney U test and the Kruskal-Wallis ANOVA test were used to compare independent group differences. The chi-square analysis was used to compare categorical variables. The logistic regression method was used to determine the risk factors that influence SD. P<0.05 was considered significant in all analyses.

Molecular analysis

Genomic DNA was extracted using the Fujifilm Quick DNA isolation kit (Fujifilm, Japan) according to the manufacturer's instructions. Genomic DNA genotyping was performed by polymerase chain reaction (PCR)-restriction fragment length polymorphism (RFLP) for DRD2 -141C Ins/Del, DRD2Taq1A, eNOS894G>T,eNOS-786T>C, using methods as described above [15-18]. TaqI for Taq1A, BstNI for -141C Ins/Del, BanII for G894T, and MspI for T-786C (Vivantis Technologies, Malaysia) enzymes used in the RFLP, and the products separated by electrophoresis on 4% agarose were gel stained with ethidium bromide.

## Results

As ASEX is used for the evaluation of SD, the presence of SD is defined as SD+ (with SD) if the total ASEX score is 19 or higher, five points on any item, or four points on any three items according to ASEX.

Approximately 74 schizophrenia and 37 bipolar disorder patients participated in the study. SD was found in 50.45% (N:56) of all patients. The rate of patients with SD was 45.9% (N: 34) in the schizophrenia group, and 59.5% (N:22) in the bipolar disorder group, and no statistically significant difference was found between the groups (p=0.179).

While no statistically significant difference was found between the groups with and without SD in terms of gender, marital status, and the duration of illness in both patient groups (p<0.05), the mean age was found to be significantly higher in SD+ groups (p=0.004). Considering all the individuals included in the study, the evaluation of education level revealed that a statistically significant difference was found between SD+ and SD- groups. The frequency of SD decreased as the level of education increased and, the mean age was found to be significantly higher in SD+ groups (Table 1).

**Table 1:** Relationship of SDs with sociodemographic data and duration of disease *: p<0.05 statistically significant difference; SD+: with sexual dysfunction; SD-: without sexual dysfunction

		All patients		P
		SD+ N (%)	SD- N (%)	
Age mean±SD		42.84 ± 8.6	36 ± 9.87	0.0001*
Gender	Woman	19 (33.9)	23 (41.8)	0.39
	Man	37 (66.1)	32 (58.2)	
Marital status	Married	48 (85.7)	41 (74.5)	0.14
	Single	8 (14.3)	14 (25.5)	
Educational status	Primary school	33 (58.9)	14 (25.5) 11 (20)	0.0001*
	Secondary school	13 (23.2)	11 (20)	
	College	8 (14.3)	19 (34.5)	
	University	2 (3.6)	11 (20)	
Disease duration (months) mean ± SD	Woman	114.37 ± 99.05	125.22 ± 100.97	0.71
	Man	147.84 ± 117.84	124.63 ± 99.25	0.42
	All	136.48 ± 112.5	124.87 ± 99.04	0.56

No statistically significant difference was found in the mean SAPS and CDSS scores between the groups with and without SD in schizophrenia patients (p=0.952, p=0.359 respectively), but the mean SANS score in the SD+ group was found to be statistically significantly higher (p=0.023).

No statistically significant difference was found between SD+ and SD- groups in the mean scores of the HAM-D and YMRS scales in bipolar disorder patients (p=0.91, p=0.86). No statistically significant difference was found between the mean prolactin values and the presence or absence of SD in the schizophrenia and bipolar disorder patient groups (p=0.479, p=0.593). When analyzed by gender, mean prolactin values were found to be significantly higher in female schizophrenia patients than in male schizophrenia patients (p=0.005).

In this study, values above 15 ng/mL according to laboratory reference values were considered as hyperprolactinemia. Hyperprolactinemia was detected in 78.6% (N:55) of the schizophrenia patients and 67.6% (N: 25) of the bipolar disorder patients. Considering all patients, there was no statistically significant difference in the presence of SD according to the presence or absence of hyperprolactinemia. The number of patients with and without SD in the hyperprolactinemia group was found to be equal.

The smoking rate was 60.81% (N:45) in the schizophrenia group and 57.14% (N: 20) in the bipolar group. Although there was no statistically significant difference between the groups with and without SD in the presence of smoking according to the patient groups, the mean amount of smoking (pack/year) was found to be significantly higher in individuals with SD in both patient groups (p=0.009 for the schizophrenia group, and p=0.02 for the bipolar disorder group).

According to the type of drug use, the patients in our study were evaluated in five groups (Table 2). There was no statistically significant difference in the presence of SD in all drug groups (p=0.224) (Table 2). There was no statistically significant difference between drug groups in terms of the mean ASEX scale subscale scores and the total scores.

**Table 2:** Rates of SD in drug groups *: p<0.05 statistically significant difference; SD+: with sexual dysfunction; SD-: without sexual dysfunction; AP: antipsychotic; AC: anticholinergic; MS: MoodStabilizer; AD: antidepressant

	All patients		
	SD+ N (%)	SD- N (%)	P
AP	20 (35.7)	23 (41.8)	0.224
AP+AC	6 (10.7)	13 (23.6)	
AP+AD	8 (14.3)	6 (10.9)	
AP+MS	14 (25)	7 (12.7)	
More than two groups of drugs	8 (14.3)	6 (10.9)	

Considering all individuals included in the study, no statistically significant difference was found between the DRD2-141C Ins/Ins and Ins/Del+Del/Del genotypes in the presence of SD and also between the frequency of the Ins and Del alleles in the presence of SD (p=0.929, p=0.73; respectively) (Table 3).

In this study, DRD2Taq1A A1/A1 homozygous numbers in schizophrenia patients were not included in the statistical calculation as there was a subject with SD+ and two subjects with SD-. A1/A1 polymorphism was never seen in the bipolar disorder group. Considering all patients, no statistically significant difference was found between DRD2Taq1A A2/A2 and Taq1A A1/A2 genotypes as well as DRD2Taq1A A1 and A2 allele frequencies in the presence of SD (p=0.532 and p=0.63, respectively) (Table 3).

**Table 3:** The relationship of allele frequencies with SD *: p<0.05 statistically significant difference; SD+: with sexual dysfunction; SD-: without sexual dysfunction; Del: deletion; DRD2: D2 dopamine receptor; eNOS: endothelial nitric oxide synthase; Ins: insertion

All patients				
		SD+	SD-	P
DRD2-141C	Ins allele frequency	105 (95.5)	102 (94.4)	0.73
	Del allele frequency	5 (4.5)	6 (5.6)	
DRD2 Taq1	A1 allele frequency	20 (18.2)	17 (15.7)	0.63
	A2 allele frequency	90 (81.8)	91 (84.3)	
eNOS-786T>C	C allele frequency	20 (17.4)	31 (28.7)	0.04*
	T allele frequency	95 (82.6)	77 (71.3)	
eNOS894G>T	G allele frequency	56 (50.9)	69 (58.5)	0.25
	T allele frequency	54 (49.1)	49 (1.5)	

Considering the eNOS-786T>C C, CT, and TT polymorphisms, there was no significant difference between the specified genotypes in having SD or not when all patients were included. When eNOS-786T>CC and T allele frequency and the presence of SD were compared, T allele frequency was found to be significantly higher in the SD+ group (p=0.044) (Table 3). The logistic regression analysis revealed that the presence of the T allele clinically increased the risk of SD compared to the presence of the C allele, although there was no statistical significance as there was an accumulation in the eNOS-786T>C TT genotype. Again, clinically, it was determined that the presence of CT (p=0.624, OR=1.474) and TT genotypes (p=0.198, OR=2.621) increased the risk of SD compared to the presence of CC genotype, and the risk difference between them was greater between CC and TT genotypes. By adding age and smoking to the logistic regression model, it was determined that the factor that increased the risk of SD (+) the most was age (Table 4).

**Table 4:** Logistic regression of CT and TT genotype by reference category CC combined with age and smoking *: p<0.05 statistically significant difference; eNOS: endothelial nitric oxide synthase; OR: odds ratio

	P	OR	95% confidence interval	
			Lower limit	Upper limit
eNOS-786T>C (CT)	0.931	0.929	0.174	4.955
eNOS-786T>C (TT)	0.529	1.668	0.339	8.201
Smoking (+)	0.288	1.581	0.679	3.679
Age	0.001*	1.079	1.031	1.129

When eNOS894G>T GG, GT, TT polymorphisms, and G and T allele frequencies were evaluated, no significant difference was found in the presence of SD (p>0.05). Logistic regression did not demonstrate statistical significance as there was an accumulation in the TT genotype. According to logistic regression, the presence of the T allele clinically compared to the presence of G allele, and the presence of GT (p=0.354, OR=1.50) and TT genotypes (p=0.069, OR=2.695) clinically increased the risk of SD compared to the presence of GG genotype. Additionally, the risk difference was found to be greater between GG and TT. Adding age and smoking to the logistic regression model, the factor that increased the risk of SD (+) the most was determined to be age (Table 5).

**Table 5:** Logistic regression of GT and TT genotype by reference category GG combined with age and smoking *: = p<0.05 statistically significant difference; eNOS: endothelial nitric oxide synthase; OR: odds ratio

	P	OR	95% confidence interval	
			Lower limit	Upper limit
eNOS894G>T (GT)	0.658	1.232	0.490	3.100
eNOS894G>T (TT)	0.099	2.589	0.835	8.023
Smoking (+)	0.156	1.834	0.793	4.237
Age	0.001*	1.077	1.029	1.127

## Discussion

Studies evaluating SD in schizophrenia and bipolar patients in Turkey are scarce. A study that evaluated SD in schizophrenia in Turkey found the rate of SD was 55.4% [19]. In a study conducted among bipolar disorder patients, the rate of SD was reported as 50% (N: 25) [3]. The rates of SD in this study are consistent with the literature [3,19].

Although this study found that the SD rate increased with increasing age in patients with schizophrenia and bipolar disorder, we think that age could be a risk factor for SD independent from the duration of the disease because no relationship was found between the duration of the disease and SD.

In this study, SD increased as the education level decreased. The negative relationship between education level and SD may be because of the better expression of the problem in individuals with higher education levels and the better management of the problem with factors such as sharing between spouses and seeking professional help.

Various results have been reported in studies looking at the relationship between prolactin and SD. In a study, serum prolactin levels were found to be significantly higher in patients with SD [20]. In another study, it was reported that there was no statistically significant difference in prolactin levels between groups with and without SD according to the ASEX scale, but there was a significant difference in serum prolactin levels between groups with and without erectile dysfunction according to the IIEF-5 scale [21]. The fact that there was no significant difference in mean prolactin values between groups with and without SD according to ASEX our study is consistent with the results of the study by Zhang et al. [21]. However, there was no difference in the presence or absence of SD in patients with hyperprolactinemia, and there was no significant difference between genders in terms of the presence of SD even though the mean prolactin values were found to be significantly higher in the female schizophrenia patient group than in the male patients; these findings indicate the importance of non-prolactin factors.

No significant difference was found in terms of the presence of smoking in the schizophrenia and bipolar disorder patient groups with SD while the amount of smoking was significantly higher in the SD group. This finding suggests that the quantity of cigarettes is important in SD.

Considering all of the patients, there was no statistically significant difference between the presence of SD or the ASEX scale total and subscale mean scores according to drug groups. In this study, the lack of a statistically significant difference in SD rates between drug groups may be because of the use of antipsychotics in all drug groups.

Possible effects of DRD2 gene polymorphisms, which are thought to be a schizophrenia susceptibility gene, and possible side effects in treatment are among the issues that have attracted attention recently [22,23].

In the literature, there is a study evaluating DRD2-141C polymorphisms in SD in patients with schizophrenia [21]. The fact that there was no difference in the mean prolactin levels and the presence of SD according to DRD2-141C polymorphisms in our study and that there was no statistically significant difference in the presence of SD according to Ins and Del allele frequency rates is inconsistent with the study of Zhang et al. [21]. The reasons such as the inhomogeneity of drug use groups and doses between studies, and the fact that Zhang et al.'s study included only male schizophrenia patients make comparisons difficult.

In the literature, there are studies on DRD2Taq1A1 polymorphisms reporting that the DRD2 Taq1A1 allele is associated with low DRD2 density and binding in the striatum [24]. It was stated that DRD2Taq1A A1 allele increases the sensitivity for antipsychotic side effects by decreasing DRD2 receptor density and binding. Our results were not consistent with this statement because DRD2Taq1A polymorphisms and allele frequencies did not influence sexual functions and mean prolactin levels. However, our results were consistent with Zhang et al.'s study [21]. More in vivo and in vitro studies are needed in this area.

NO is a potent vasodilator in penile and clitoral tissue and is produced by three nitric oxide synthase (NOS) isoenzymes. nNOS mediates the release of NO in peripheral nitrergic cells, and eNOS affects the smooth muscle relaxation of cavernous tissue [25].

Considering the nNOS polymorphisms, a statistical comparison could not be made because only one of the patients participating in the study had TT polymorphism, and the remaining patients had CC polymorphisms.

Research suggests that eNOS-786T>C polymorphism was found to reduce the serum NO level significantly by affecting the eNOS gene promoter activity, while the eNOS894G>T polymorphism can reduce the eNOS enzyme activity [26,27].

In the literature, there is a study that did not detect a relationship between eNOS-786T>C polymorphism and SD [21]. Our study revealed that the eNOS-786T>C T allele frequency ratio was found to be significantly higher than the C allele frequency ratio in patients with SD, which indicates that the T allele may predispose to SD.

Considering the eNOS894G>T polymorphisms, there is a study reporting that the T allele is associated with erectile dysfunction and, there is a study that did not find a significant relationship [21,28]. According to the logistic regression analysis in our study, clinically, the higher rates of SD in patients with the T allele compared to the G allele may indicate that the T allele is a risk factor for SD.

The various results of studies evaluating the relationship between eNOS polymorphism and SD may be because of factors such as the regulation of eNOS by many stimuli at the transcriptional, posttranscriptional, and posttranslational levels as well as the fact that the studies were conducted in different patient and gender groups and different designs [29].

One limitation of this study is the absence of a healthy control group. Although the sample size of the study was sufficient, it was observed that there was an accumulation of patients in some drug groups when attempting to evaluate SDs according to drug groups. This is another limitation of the study.

In conclusion, the rates of SD in patients with schizophrenia and bipolar disorder in this study confirm the necessity of considering SD, which may cause treatment dropouts and deterioration in the quality of life of these patient groups. The high rates of SD in patients with low education levels and high amounts of smoking indicate the importance of intervening in these modifiable factors. On the other hand, the high rate of SD in patients with the eNOS -786T>C T allele indicates the importance of conducting new studies investigating the factors affecting the enzyme activity in this genotype. There is a need for more studies on eNOS genotypes and enzyme activities in this area.

## Conclusions

In this study, the high rates of SD in patients with schizophrenia and bipolar disorder, and the presence of modifiable factors that influence the presence of SD, suggest that SD should be given more attention in these patient groups. On the other hand, the high rate of SD in patients with the eNOS -786T>C T allele indicates the importance of conducting new studies investigating the factors affecting the enzyme activity in this genotype. There is a need for more studies on eNOS genotypes and enzyme activites in this area.
